# Craniotomy hematoma was removed from the bone flap and multiple holes were drilled on the bone flap: A case report

**DOI:** 10.1097/MD.0000000000033781

**Published:** 2023-05-26

**Authors:** Wei Zhu, Lei Ma, Tuo Li, Hongguang Chen

**Affiliations:** a Department of Neurosurgery, Yantai Yuhuangding Hospital, Yantai, China.

**Keywords:** case report, CSDH, neurosurgery

## Abstract

Chronic subdural hematoma (CSDH) is a common disease in neurosurgery department. Burr-hole drainage is the main surgical treatment. And the recurrence rate is as high as 25%.

**Case Report:**

In this case, a male patient with CSDH in the left frontotemporal parietal region underwent 2 drilling and drainage operations in the local hospital, but the hematoma recurred after operations. Being unable to bearing the repeated and progressive aggravation of headache, he came to our hospital for treatment. After considering the comprehensive situation, we use a new surgical method, removal of hematoma by drilling multiple holes in the lateral skull, to cure the patient.

**Conclusions:**

We get inspirations from the treatment of moyamoya disease surgery, through the bone holes the scalp forms many “meat column” like structures which have powerful capability in absorption, so the scalp could deep into the hematoma, then the CSDH could be cured. Provide a new surgical method for the treatment of refractory CSDH.

## 1. Introduction

Chronic subdural hematoma (CSDH) is a prevalent kind of intracranial hematoma with an incidence of 1 to 13.1 per 100,000 per year in the neurosurgery department.^[1–3]^ Sixty to eighty percent of older individuals with a history of moderate head injury are more prone to develop CSDH which accounts for about 10% of intracranial hematomas. CSDH, which locates between the dura mater and the arachnoid membrane, oppresses the brain directly or provokes increased intracranial pressure, thereby causing corresponding clinical symptoms gradually in 2 to 3 weeks after trauma.^[4]^ Even though the initial clinical signs are minor, the symptoms quickly worsen when the hematoma volume reaches a particular level. Although some patients can take atorvastatin and low dose-dexamethasone for treatment,^[5]^ there are still quite a considerable amounts of patients who need surgical treatment. There are 2 main ways to remove the hematoma, namely burr hole irrigation or craniotomy. In addition, it is inevitable for the rate to recur that it could approach 25%.^[5]^ Furthermore, CSDH tends to occur in the elderly, which means a high risk of peri-operational infection, pneumonia, and high-surface-tension pulmonary edema. We experienced a case of refractory CSDH that achieved ideal outcomes. In this case, we performed a craniotomy to remove a subdural hematoma and drilled multiple holes in the bone flap on the patient under general anesthesia.

## 2. Case report

A 66-year-old man was hospitalized for headache and dizziness which had lasted for 3 months and aggravated a day ago. Three months ago, the patient complained of a headache, dizziness, and shaky gait without any clear cause. In April 30th, 2019, a local hospital did a cranial CT scan that revealed a CSDH with a volume of 30 mL in the left frontotemporal parietal area. At the local hospital, the patient underwent the minimally invasive trepanation and drainage on the May 5th and then the symptoms relieved. Thirteen days following the procedure, the patient experienced terrible headaches and vertigo once more. Reexamination of the head CT revealed that a 40 mL CSDH had returned.

On June 16th, 2019, the patient arrived at our hospital complaining of a terrible and incapacitating headache. Physical examination revealed that the patient was aware and had normal limb mobility with a GCS score of E4V5M6.

**Figure 1.**
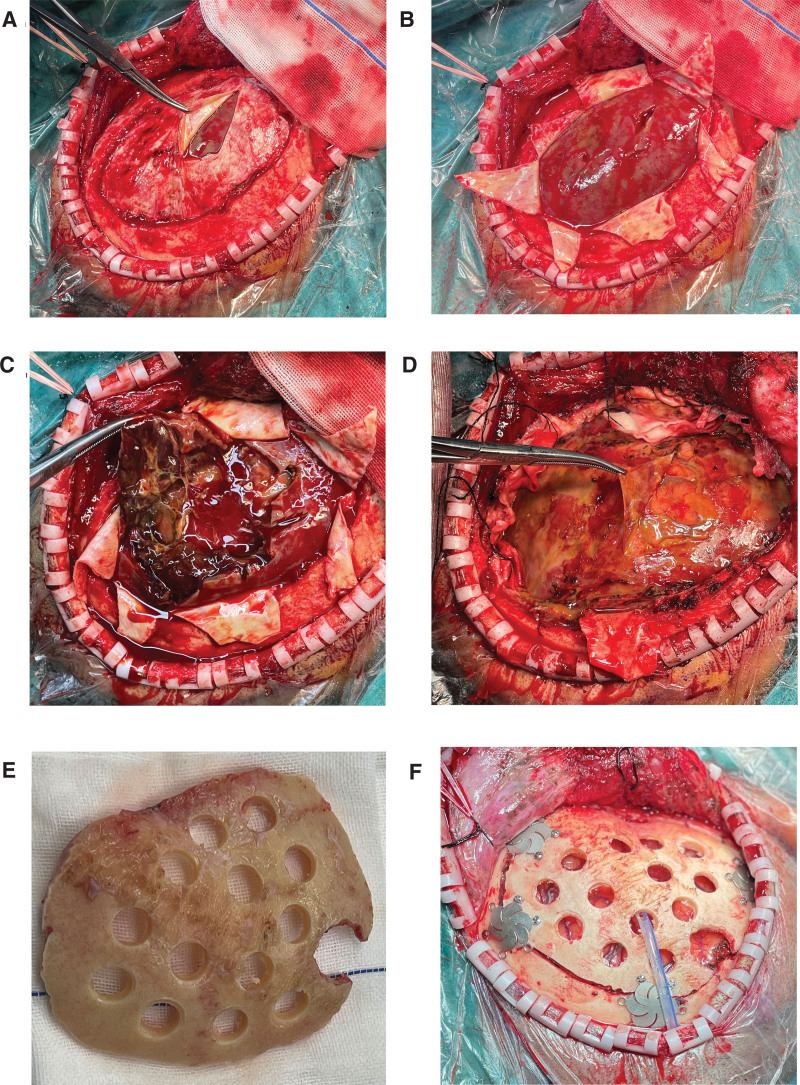
Intraoperative findings and the treatment of bone flap. (A and B) Subdural hematoma envelope could be clearly observed after craniotomy. (C and D) Organized chronic subdural hematoma and visceral membrane of hematoma could be found after the surgery. (E and F) Treatment of intraoperative bone flap.

**Figure 2.**
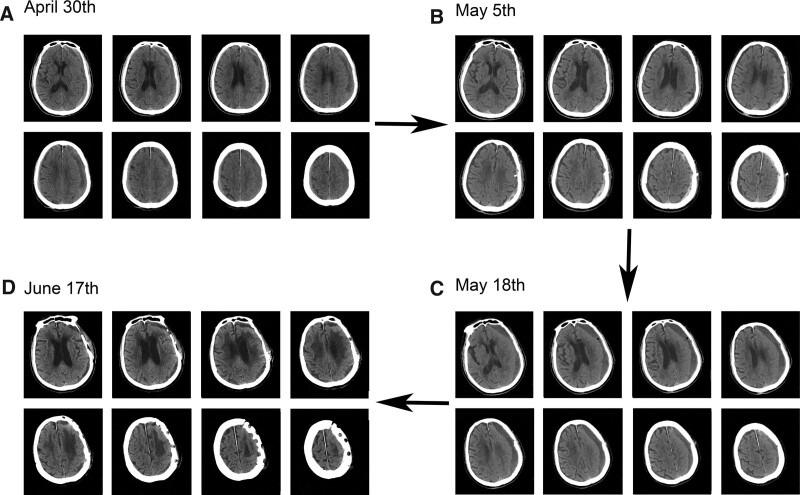
Treatment process and imaging features. (A) Chronic subdural hematoma emerged on the left frontotemporal-parietal lobe on initial admission. (B) The volume of chronic subdural hematoma significantly reduced after the first drainage. (C) Chronic subdural hematoma recurred. (D) After multiple bone flap drilling, chronic subdural hematoma was thoroughly removed.

We performed a craniotomy to remove a subdural hematoma, drilled multiple holes in the bone flap under general anesthesia. Intraoperatively, the hematoma was observed as meconium which had a thick envelope (Fig. 1A–D). Besides, it was confirmed that the volume of the hematoma matched the CT results (Fig. 2). The goal of surgery was to completely remove the hematoma and its envelope; following the recovery of the bone flap, the loss of the skull could be avoided. Then through several bone holes the scalp could form several flesh-like structures (Fig. 1E and F). Therefore, the scalp is tightly bound to the brain tissue through this column of flesh. The remaining hematoma was absorbed by the scalp vessels’ absorption function, preventing the recurrence of CSDH. Following surgery, the patients’ clinical problems gradually subsided and then vanished. Three years of patient monitoring passed with no recurrence.

## 3. Discussion

There are 3 treatments were considered for this patient: Trepanation and drainage of CSDH again; Neuroendoscopic hematoma and capsule removal; Hematoma and capsule removal of bone flap decompression.

Two main components of the potential risk factors for CSDH recurrence after surgery are being taken into consideration. One factor is the patient individual traits, such as advanced age, brain atrophy, a propensity for bleeding, liver and renal disease, alcoholism, diabetes, epilepsy, and low cranial pressure. Another is the CSDH disease itself, which manifests as high and low mixed density in CT scans, hematomization, and compartmentalization in the hematoma cavity.^[6]^ Additionally, the increased fibrin degradation product in the hematoma fluid and the local fibrinolysis hyperactivation may cause the capillaries and venules in the hematoma outer membrane to bleed slowly and continuously. Therefore, the pace of plasma exudation and capillary neovascularization of the hematoma, which exceeds the rate of liquefaction and re-absorption of the hematoma, as well as the difficulty of angiogenesis, are directly associated to the rise and recurrence of refractory subdural hematoma.^[7]^ Additionally, characteristics associated with surgery, such as insufficient intraoperative and postoperative drainage and gas buildup in the hematoma cavity, are strongly correlated with CSDH recurrence. In conclusion, the primary causes of CSDH recurrence include advanced age-related brain atrophy, hematoma division and numerous chambers, capsule formation and surgical capsule residue, hematoma mechanization, and excessive inflammatory response to the hematoma outer membrane.^[8]^

In our opinion, the 3 methods mentioned above have some shortcomings: Even though the local hospital twice implemented the drainage of small holes, the persistent subdural hematoma continued to recur; Because the hematoma envelope is too extensive to remove completely, hematoma clearance and Hematoma envelope resection are challenging utilizing neuroendoscopy. Bleeding or recurrence may occur in the postoperative period because it can sometimes be difficult to halt the bleeding in the leftover capsule; The boneless disc and hematoma clearing can be extremely traumatic procedures. Then we performed a craniotomy to remove a subdural hematoma and drilled multiple holes in the bone flap. This avoided the recurrence of the hematoma and preserved the bone flap.

## 4. Conclusion

Atorvastatin is a reliable and conservative medicine for CSDH.^[9]^ However, for quite an amount of people who suffer from excruciating headaches, mental health issues, or even a chronic cerebral hernia, surgery may be the best option. The primary surgical procedure for CSDH at the moment is skull drilling, flushing, and drainage, which can produce satisfactory results. It straightforward and risk-free to perform this procedure. The recurrence rate following surgery, according to clinical investigations, is reported to be between 3.7 and 30 percent, and refractory CSDH is one of the significant problems that may be fatal.^[10]^ For recurrent refractory CSDH, the current surgical methods are as follows: Neuroendoscopic hematoma removal; Craniotomy for removal of hematoma. Both methods mentioned above have merits and demerits. We developed coupled dura mater inversion with multiple bone perforations for a refractory CSDH, which was inspired by the surgical procedure for moyamoya. We make channels so the hematoma can be in contact with the scalp. Then, by using the mature blood vessels on the scalp resorption function, CSDH may be digested more quickly while the likelihood of recurrence may diminish. We provide a novel concept and demonstrate that combined dura mater inversion with several bone perforations for refractory chronic subdural hemorrhage is achievable.

Following surgery, the patient clinical symptoms gradually subsided and then vanished, allowing them to resume their usual lives.

## Author contributions

**Conceptualization:** Wei Zhu.

Data curation: Wei Zhu.

Investigation: Hongguang Chen.

Methodology: Tuo Li.

Project administration: Tuo Li.

Validation: Wei Zhu, Hongguang Chen.

Writing – review & editing: Lei Ma.
